# Primary sclerosing cholangitis associated with CREST (calcinosis, Raynaud phenomenon, oesophageal dysmotility, sclerodactyly and telangiectasia) in an elderly woman: a case report

**DOI:** 10.1186/s13256-015-0747-9

**Published:** 2015-11-25

**Authors:** Alice Powell, Julian McNeil

**Affiliations:** 1 The Queen Elizabeth Hospital, Adelaide, SA Australia; 2 University Department of Medicine, Modbury Hospital, Adelaide, SA Australia

**Keywords:** Bosentan, Cholestasis, CREST, Liver toxicity, Primary sclerosing cholangitis

## Abstract

**Introduction:**

CREST (calcinosis, Raynaud phenomenon, oesophageal dysmotility, sclerodactyly, and telangiectasia) syndrome comprising calcinosis cutis, Raynaud phenomenon, esophageal dysmotility, sclerodactyly and telangiectasia and primary sclerosing cholangitis are both chronic fibrotic diseases but the association between them is extremely rare. While primary sclerosing cholangitis has been associated with diffuse cutaneous scleroderma, the association with limited cutaneous scleroderma or CREST has not been previously reported in the literature. This case report illustrates the association between CREST and primary sclerosing cholangitis.

**Case presentation:**

We report the case of an 84-year-old Asian woman with a long history of CREST who was admitted with abdominal pain, fatigue and progressive derangement of her liver enzymes. This was initially thought to be secondary to her bosentan therapy for pulmonary hypertension but it persisted despite bosentan being ceased. Primary sclerosing cholangitis was subsequently diagnosed on magnetic resonance cholangiopancreatography and she was referred to a hepatologist for treatment.

**Conclusions:**

This case highlights the need to consider primary sclerosing cholangitis in patients with CREST who present with abdominal symptoms and deranged liver enzymes when other causes have been excluded. Relevant differential diagnoses for this presentation, which can be difficult to exclude, include immunoglobulin G4-associated cholangitis and antimitochondrial antibody negative primary biliary cirrhosis. It is of particular significance to rheumatologists and gastroenterologists but has broader relevance to all medical specialists involved in the care of patients with CREST.

## Introduction

Primary sclerosing cholangitis (PSC) is a rare idiopathic chronic progressive liver disease resulting in fibro-obliterative inflammation of the hepatic bile ducts and ultimately cirrhosis and liver failure [1]. It is more frequent in men and strongly associated with inflammatory bowel disease [1, 2]. The liver disease usually associated with scleroderma is primary biliary cirrhosis (PBC) with approximately 15 % of patients with PBC reported to have scleroderma [3]. The association was first made by Murray-Lyon *et al.* in the 1970s [4]. In contrast, the relationship between PSC and scleroderma is extremely rare [3] with only one case report, to the best of our knowledge, of PSC and diffuse cutaneous scleroderma [2]. We describe a case of PSC occurring in a patient with CREST (calcinosis cutis, Raynaud phenomenon, esophageal dysmotility, sclerodactyly and telangiectasia) syndrome, which is a type of limited cutaneous scleroderma.

**Fig. 1:**
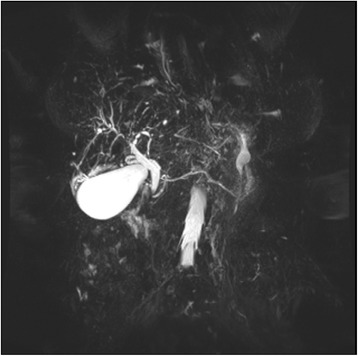
Magnetic resonance cholangiopancreatography demonstrating prominent common bile duct and prominent irregular hepatic ducts

## Case presentation

An 84-year-old Asian woman was electively admitted to our hospital for investigation of a 6-month history of liver function test (LFT) derangement, abdominal pain, fatigue and weight loss. The results of her LFTs were as follows: alkaline phosphatase 953 U/L, gamma glutamyl transpeptidase 1164 U/L, alanine aminotransferase 81 U/L, aspartate aminotransferase 89 U/L, bilirubin 13 μmol/L and albumin 33 g/L. She had experienced Raynaud’s phenomenon for 50 years and had a confirmed diagnosis of CREST complicated by pulmonary hypertension, for which she had been treated with bosentan for the past 7 years. Her abdominal pain was initially attributed to esophageal dysmotility associated with her CREST and had improved with pantoprazole.

The most frequent severe side effect of bosentan is liver toxicity, typically manifested by a transaminasemia [5]. Of interest, animal models have demonstrated that bosentan may also cause cholestatic liver injury through inhibition of the bile salt export pump (BSEP) [6]. Our patient’s LFT derangement was initially attributed to bosentan and this was decreased and then changed to macitentan which does not interfere with BSEP [6]. However, her LFT derangement gradually worsened despite these measures and therefore other causes were sought. There were no other new medications, her hepatitis serology was negative and there was no evidence of cholecystitis, cholelithiasis or biliary obstruction on ultrasound. Computed tomography (CT) of her abdomen was also unremarkable. An autoimmune screen revealed positive antinuclear antibody consistent with CREST, antimitochondrial antibody and other liver autoantibodies negative. Immunoglobulin levels including immunoglobulin G4 (IgG4) were normal. Tumor markers including cancer antigen 19–9 (CA 19–9) and carcinoembryonic antigen (CEA) were similarly normal. She proceeded to magnetic resonance cholangiopancreatography (MRCP) which demonstrated a prominent common bile duct and prominent irregular central hepatic ducts with areas of beading and narrowing in both lobes favoring stricturing and PSC (Fig. 1).

A liver biopsy was not pursued given her age and comorbidities, in particular her pulmonary hypertension. While this may have provided useful additional information particularly in the exclusion of other differential diagnoses, findings can be nonspecific [7]. In addition, a biopsy is generally not required for the diagnosis of PSC where cholestatic biochemical abnormalities and characteristic changes on cholangiography are present.

## Discussion

PSC is a complex heterogeneous probably immune-mediated disease with an unpredictable clinical presentation and course. Diagnosis is made in patients with a cholestatic biochemical profile and characteristic bile duct changes on cholangiography (MRCP or endoscopic retrograde cholangiopancreatography, ERCP) where secondary causes of sclerosing cholangitis have been excluded [1]. Typical symptoms include right upper quadrant abdominal pain, fatigue, pruritus and weight loss. Autoantibodies have no role in the routine diagnosis of PSC but antimitochondrial antibody is usually negative whereas it is positive in PBC [1]. Abdominal ultrasound and CT findings are usually nonspecific and ERCP has been regarded as the gold standard for diagnosis. However, MRCP has comparable diagnostic accuracy and is noninvasive without the potential for pancreatitis and bacterial cholangitis [1]. In a meta-analysis of six studies, the sensitivity and specificity of MRCP for diagnosing PSC were 86 and 94 % respectively [8]. In the presence of an abnormal cholangiogram, a liver biopsy is not required to establish a diagnosis of PSC. No drug therapy has been conclusively proven to alter the natural history of this disorder and liver transplantation is the treatment of choice for advanced liver disease [1].

PSC should be differentiated from secondary causes of sclerosing cholangitis and IgG4-associated cholangitis. Another potential differential diagnosis for this case was antimitochondrial antibody negative PBC as antimitochondrial antibody may be negative in less than 10 % of cases [9]. However, patients with PBC should have a normal biliary tree on cholangiography [9]. Secondary causes of PSC were ruled out on the basis of our patient’s history, physical examination and imaging studies. Of importance, there was no dominant stricture on MRCP. IgG4-associated cholangitis is more difficult to differentiate and it is possible that IgG4-associated cholangitis, autoimmune pancreatitis and PSC are different manifestations of the same disease [10]. This is, however, an important distinction as IgG4-associated cholangitis may be glucocorticoid responsive [10], while no effective medical therapy is available for PSC. This diagnosis was thought less likely in our patient’s case as IgG4-associated cholangitis rarely occurs in the absence of pancreatitis, for which there was no evidence, and her IgG4 level was normal [10].

## Conclusions

Both scleroderma and PSC are fibrotic diseases with widespread connective tissue disturbance occurring in CREST and abnormal collagen deposition in the bile duct epithelium. While PSC has been reported to occur in diffuse cutaneous systemic sclerosis, this is the first case report of PSC occurring with limited cutaneous systemic sclerosis.

## Consent

Written informed consent was obtained from the patient for publication of this case report and any accompanying images. A copy of the written consent is available for review by the Editor-In-Chief of this journal.

## Abbreviations

BSEP: bile salt export pump
CREST: calcinosis cutis, Raynaud phenomenon, esophageal dysmotility, sclerodactyly and telangiectasia
CT: computed tomography
ERCP: endoscopic retrograde cholangiopancreatography
IgG4: immunoglobulin G4
LFT: liver function tests
MRCP: magnetic resonance cholangiopancreatography
PBC: primary biliary cirrhosis
PSC: primary sclerosing cholangitis
